# NcROP2 deletion reduces *Neospora caninum* virulence by altering parasite stage differentiation and hijacking host immune response

**DOI:** 10.3389/fimmu.2025.1617570

**Published:** 2025-08-12

**Authors:** Rafael Amieva, Montserrat Coronado, Jessica Powell, David Arranz-Solís, Musa A. Hassan, Esther Collantes-Fernández, Luis Miguel Ortega-Mora, Pilar Horcajo

**Affiliations:** ^1^ SALUVET Group, Animal Health Department, Faculty of Veterinary Sciences, Complutense University of Madrid, Madrid, Spain; ^2^ Division of Infection and Immunity, The Roslin Institute, University of Edinburgh, Edinburgh, United Kingdom

**Keywords:** *Neospora caninum*, virulence factor, NcROP2, CRISPR/Cas9, BALB/c, bovine macrophages, transcriptome

## Abstract

**Introduction:**

*Neospora caninum* is an apicomplexan parasite responsible for bovine neosporosis, a major cause of abortion in cattle worldwide. *N. caninum* rhoptry protein 2 (NcROP2) has been identified as an essential factor in host cell invasion and parasitophorous vacuole formation, making it a potential target for disease control strategies.

**Methods:**

In this study, we generated *NcRop2* knockout (*NcΔROP2*) mutants using CRISPR/Cas9 technology to assess their role in parasite virulence.

**Results:**

In a pregnant mouse model, *NcΔROP2* parasites exhibited reduced virulence, as indicated by increased neonatal survival rates and lower parasite burden in the brain and attenuated clinical signs in the dams compared to the wild-type (Nc-Spain7) parental strain. Additionally, the *NcΔROP2* mutants exhibited impaired proliferation and significantly induced the expression of interferon-stimulated genes in bovine monocyte-derived macrophages infected *in vitro* for 60 hours. Transcriptomic analysis further revealed a shift in parasite gene expression, with an upregulation of stress-related and bradyzoite markers. Functional assays confirmed that *Nc*Δ*ROP2* parasites were less susceptible to IFN-γ-mediated inhibition and displayed an enhanced ability to convert to the semi-dormant bradyzoite stage.

**Discussion:**

These findings highlight NcROP2 as a key virulence factor involved in immune evasion and parasite proliferation, providing new insights into *N. caninum* infection pathogenesis and potential avenues for vaccine development.

## Introduction

*Neospora caninum* is an obligate intracellular apicomplexan parasite closely related to *Toxoplasma gondii* and the etiological agent of bovine neosporosis, a significant cause of abortion in cattle worldwide (1). Transmission in cattle may occur through the ingestion of sporulated oocysts (horizontal transmission) or transplacentally during pregnancy (vertical transmission) from infected dams to fetuses. The infection outcome ranges from abortion to the birth of healthy yet congenitally infected calves (2). Currently, there is a lack of effective treatment and preventive control measures. Therefore, understanding the molecular mechanisms that control *N. caninum* virulence is crucial to developing effective control strategies. The initial host cell recognition is mediated by parasite surface antigens, whereas the actual invasion process is dependent on specific molecular interactions between host receptors and parasite ligands that are secreted from a set of organelles (micronemes, rhoptries and dense granules) at the apical complex. Several of these secreted proteins have been identified as key virulence factors, including the rhoptry proteins (ROP) NcROP16 (3), NcROP5 (4) and NcROP40 (5, 6); and the dense granule proteins (GRA) NcGRA7 (5–7), and NcGRA6 (8).

Among the various proteins involved in parasite pathogenicity, ROPs play a crucial role in the parasite lytic cycle and its interaction with the host. For example, in the closely related *T. gondii* parasites, the ROP2 family constitutes a major group of rhoptry proteins characterized by their structural similarity to protein kinases, though only a subset retains the essential catalytic residues required for enzymatic activity (9). These proteins play diverse roles in parasite-host interactions, with some members, such as ROP16, translocating to the host cell nucleus to modulate signaling pathways, while others primarily localize to the parasitophorous vacuole membrane (PVM), where they contribute to vacuole formation and immune evasion (10). By manipulating host cell processes, ROP2 family proteins facilitate parasite survival and replication. Functional studies have demonstrated that depletion of TgROP2 leads to impaired host cell invasion and a significant reduction in virulence in murine models (11). In *N. caninum*, NcROP2 is part of the apical complex and is essential for host cell invasion and the formation of the parasitophorous vacuole, a structure critical for parasite survival and replication within host cells (12). Synthesized as a pre- protein, NcROP2 matures into a protein that localizes to rhoptry bulbs and associate with the parasitophorous vacuole membrane (12). Additionally, it is present on the surface of extracellular parasites, suggesting a dual role as both an intracellular and extracellular antigen (13).

Beyond its role in parasite cycle, NcROP2 has been extensively studied for its immunoprotective properties against neosporosis. Vaccination with recombinant NcROP2 has been shown to reduce mortality and cerebral infection in mouse models, highlighting its potential as a protective antigen (14, 15). Moreover, its efficacy is enhanced when combined with other antigens, such as *N. caninum* microneme (MIC) 1 and 3, or NcROP40, leading to significant reductions in cerebral infection and vertical transmission in mice (15, 16). However, there are critical gaps in the current knowledge of the molecular mechanisms that control NcROP2-mediated virulence and disease pathogenesis.

In the present study we investigated the role of Nc*ROP2* in *N. caninum* virulence. To do this, NcROP2 knockout strain was generated by deleting the ROP2 gene in the virulent Nc-Spain7 isolate using the CRISPR/Cas9 technology and its virulence phenotype characterized in pregnant mice. Additionally, we phenotype the impact of deleting ROP2 on parasite growth and host transcriptome modulation in bovine monocyte-derived macrophages (BMDMs).

## Materials and methods

### Ethics statement

Animal procedures were approved by the Animal Welfare and Experimentation Committee of the Complutense University of Madrid and the Animal Protection Area of the Community of Madrid, Spain (PROEX 66.7/20 and 064/19), adhering to the appropriate guidelines.

The use of genetically modified organisms was approved by the Genetically Modified Organisms Committee, and its manipulation was adjusted to that described in the current legislation (Law 9/2003; Royal Decree 178/2004; Directive 2009/41/UE).

### Parasite culture

*Neospora caninum* tachyzoites were maintained in MARC145 cells cultures as previously described (6). Parasites were passaged onto new cell monolayers every 3 days. Tachyzoites used for *in vivo* and *in vitro* assays were recovered from flasks, when the majority of the parasites were still intracellular, and a similar passage number was used for all strains (20–25 passages). Tachyzoites used for BMDMs and for human foreskin fibroblast cells (HFF) infection were purified using PD-10 Desalting Columns (GE Healthcare, Chicago, USA), as previously described (6). The quantity and viability of tachyzoites were determined by Trypan blue exclusion followed by direct counting in a Neubauer chamber.

### Generation of knockout and complemented strains

The highly virulent *N. caninum* isolate Nc-Spain7 (wild-type strain, WT) was used to generate the *N*c*Rop2* knockout (*NcΔROP2*) strain using the CRISPR/Cas9 system as previously described (17). Briefly, guide RNAs (gRNAs) targeting the 5’ and 3’ ends of the *NcRop2* (ToxoDB ID NCLIV_001970) coding regions were designed *in silico*. Subsequently, gRNA sequences were introduced in the *BsaI* cloning site of the pSS013-Cas9 plasmid (pU6, Addgene plasmid #52694) (18). The pLoxP-mCherry-DHFR plasmid (Addgene plasmid #70147), carrying the dihydrofolate reductase–thymidylate synthase (DHFR-TS) gene that confers resistance to pyrimethamine, was used as a donor template. Both the gRNA-containing plasmids and the *NotI*-linearized mCherry-DHFR plasmid (5:1 insert:gRNA molar ratio) were co-transfected by electroporation into approximately 3×10^7^ tachyzoites of the Nc-Spain7 isolate. At 24 h post transfection, knockout parasites were selected in media supplemented with 10 μM pyrimethamine (Sigma-Aldrich, St. Louis, MO, USA) for a minimum of three passages. Subsequently, cloning by limiting dilution was performed and two clones selected for phenotyping in mice (see Virulence assessment in BALB/c murine model for congenital and cerebral neosporosis section).

To generate a complemented strain of *NcΔROP2*, the coding region of *NcRop2* gene, including 1000 bp upstream and downstream of the start and stop codons, respectively, was amplified. In addition, homology arms of approximately 800 bp upstream and downstream of the CRISPR/cas cleavage site at the uracil phosphoribosyl-transferase (UPRT) gene (NCLIV_056020) were included to enhance transfection efficiency. The resulting amplicon was inserted into the multiple cloning site of the universal pUC19 plasmid, generating the pUC19-NcROP2 plasmid. Transfection was performed with a *KpnI*-linearized version of this plasmid and a pU6 plasmid containing a gRNA sequence targeting the 5’ end of the UPRT at a molar ratio of 1:5 (gRNA:insert). Complemented parasites were selected in media supplemented with 15 μM 5-fluorodeoxyuridine (FUDR, Sigma-Aldrich, St. Louis, MO, USA), and single clones obtained by limiting dilution.

Confirmation of the correct integration of DHFR-TS into the *NcROP2* locus in the KO strains, as well as the correct deletion and insertion of the exogenous copy of *NcRop2* into the UPRT locus in the complemented strains was assessed by PCR. DNA from individual clones was extracted using the Maxwell^®^ 16 Cell LEV DNA Purification Kit (Promega, Madison, WI, USA), and PCR reactions were performed using Taq DNA polymerase (Ecogen, Madrid, Spain) in a final volume of 25 μL, following manufacturer’s recommendations. All primers used are listed in **Supplementary Table S1**. In addition, the presence or absence of NcROP2 expression was confirmed by immunofluorescence assay (see “Immunofluorescence staining” section).

### Virulence assessment in BALB/c murine model for congenital and cerebral neosporosis

The virulence of *NcRop2* knockout parasites was evaluated in the well-established BALB/c model for congenital and cerebral neosporosis (19). This model is capable of detecting differences in virulence among *N. caninum* isolates (20). BALB/c mice (8-week-old) were procured from Janvier Labs (Laval, France) and housed in a room under a standard day/night cycle with *ad libitum* access to food and water. Animals were used for experimentation after 15 days of acclimatization. Induction of pregnancy in female mice was achieved through estrus-synchronization by the Whitten effect (21), and were mated for 96 h by housing one male with two females. Day 0 of pregnancy was defined as the first day females were housed with males. Subsequently, female mice were randomly distributed in 5 groups (20 females per group) and subcutaneously challenged at mid-gestation (days 7–10 of gestation) with 10^5^ tachyzoites/mouse, using two different KO clones (*NcΔROP2* #8 and *NcΔROP2* #42*)*, the complemented strain (*NcΔROP2*::*ROP2*, derived from clone *NcΔROP2* #42), the WT parental (Nc-Spain7) strain or PBS (unchallenged group). Mice were weighed between days 15 and 18 post-mating to confirm pregnancy, and pregnant mice were reallocated individually for delivery, whereas non-pregnant mice were housed in groups. Monitoring of clinical signs in non-pregnant mice or dams and their offspring was carried out daily until day 30 post-infection (pi) or 30 postpartum (pp), respectively. Briefly, the presence of clinical signs compatible with neosporosis were recorded using a scale with a score of 0 (no observable alterations), 1 (rough hair coat), 2 (rounded back), 3 (severe weight loss) or 4 (nervous signs), according to the description made by Pastor-Fernández et al. (15). As a humane endpoint, mice showing a body weight loss greater than 20% and nervous clinical signs were euthanized to prevent undue suffering. Non-pregnant mice were euthanized on day 30 pi in a CO_2_ chamber followed by cervical dislocation, while dams and offspring were euthanized at 30 days pp. Serum and brain samples of all animals were collected and stored at −80°C for subsequent analysis.

For the *N. caninum* congenital model, data on fertility rate (percentage of pregnant mice), litter size (number of pups delivered per dam) and neonatal mortality (number of deceased pups from day 2 to 30 pp) were recorded throughout the experiment. The model for cerebral neosporosis was performed with the dams and non-pregnant mice at the chronic stage of infection (day 30 pp or 30 pi, respectively) by determining the parasite burden in the brain (see section “DNA extraction and qPCR parasite quantification”). *Neospora caninum-*specific IgG1 and IgG2 levels were determined in serum from infected mice by ELISA as described below.

### Proliferation assay in naïve bovine macrophages

We used bovine monocyte derived macrophages (BMDMs) to investigate the proliferation dynamics of *N. caninum* strains in a relevant bovine innate immune cell (5). BMDMs were obtained from peripheral blood drawn from a healthy adult cow as previously described (22). Briefly, peripheral blood mononuclear cells (PBMCs) were isolated through density gradient centrifugation with Histopaque 1077 (Sigma-Aldrich, St. Louis, MO, USA). Subsequently, monocytes were isolated using mouse anti-human CD14 antibodies linked to microbeads (Miltenyi Biotec Ltd., San Diego, CA, USA), following the manufacturer’s instructions. Monocytes were seeded in 6-well plates at a density of 3×10^6^ cells/well and cultured in RPMI 1640 medium (Sigma-Aldrich, St. Louis, MO, USA) supplemented with 10% heat-inactivated fetal calf serum (FCS), 50 μg/ml gentamicin, 2mM L-glutamine, 50 μM β-mercaptoethanol and 20 mM HEPES. Additionally, 100 ng/ml GM-CSF (Kingfisher Biotech Inc, St. Paul, MN, USA) was added to promote monocyte differentiation. After an incubation period of 5 days, BMDMs were recovered and re-seeded at a density of 3× 10^6^ cells/well or 3× 10^5^ cells/well in a 6-well or 24-well culture plate, respectively (22).

The proliferation kinetics of *NcΔROP2* and Nc-Spain7 were determined in BMDMs by quantifying the number of tachyzoites at specific times of the lytic cycle by quantitative PCR (qPCR). Infected BMDMs were harvested and re-seeded for 24 h to acclimatize prior to infection. Subsequently, the BMDMs were inoculated with freshly syringe-lysed parasites at a multiplicity of infection (MOI) of 0.5. Non-infected MDMs were included as controls. At different time points during the lytic cycle of *N. caninum* (8, 24, 48, 60 and 72 hours pi), BMDMs were collected by adding 180 μl of lysis buffer (Qiagen, Germany) and 20 μl of proteinase K (Qiagen, Germany) to each well, transferred into DNAse free 1.5 ml tubes, and frozen at −80°C until used for DNA extraction for parasite quantification. All analyses were performed in 6 biological replicates obtained from 2 independent experiments, each separated by a minimum of 2 weeks.

In parallel, replicates of BMDM cultures grown on coverslips were identically infected and fixed at the same time points. Fixed cells were labelled by single immunostaining (as described below) to microscopically study the proliferation kinetics. In addition, vacuole sizes and cell infection rates (cIR: percentage of cells infected with at least one tachyzoite) were determined at 48 hours pi, when tachyzoites are still largely intracellular by immunofluorescence (see “Immunofluorescence staining” section). At least five fields were observed per coverslip.

### DNA extraction and qPCR parasite quantification

To determine the parasite burden in the brain of infected dams, DNA from brain tissue samples (50 to 100 mg) was extracted using the Maxwell^®^ 16 Mouse Tail DNA Purification Kit (Promega Madison, WI, USA) and DNA concentration was determined by spectrophotometry using a nanophotometer (NanoPhotometer^®^, Implen GmbH, Munich, Germany). DNA was extracted from BMDMs using the DNeasy Blood & Tissue Kit (Qiagen, Germany) following manufacturer’s instructions.

For all samples, parasite quantification was performed using the 7500 Fast Real-Time PCR System (Applied Biosystems, Foster City, CA, USA). The Nc5 region was used to quantify parasite DNA and the 28S rRNA gene was used to quantify host DNA in mice (23). Parasite burden was determined using a standard curve of 10^–1^ to 10^5^ tachyzoites, and subsequently normalization to the host DNA. All primers used are listed on **Supplementary Table S1**.

### Immunofluorescence staining

To evaluate the presence of NcROP2 in the KO and complemented strains, immunofluorescence test (IFAT) was performed following the protocol by Pastor-Fernández et al. (13) with minor modifications. Infected MARC-145 cells were washed three times with PBS and fixed using ice-cold methanol for ten minutes. Subsequently, cells were blocked and permeabilized in PBS with 3% BSA and 0.25% Triton-X 100 for 45 minutes at 37°C. Cultures were then incubated with the monoclonal antibody α-NcSAG1 (1:250) (24) as a surface marker and polyclonal antibody α-ROP2 (1:100) (13) for 1 h at 37°C. As secondary antibodies, Alexa Fluor 594-conjugated goat anti-mouse IgG and Alexa Fluor 488-conjugated goat anti-rabbit IgG (Life Technologies, Carlsbad, CA, USA) at a dilution of 1:1000 were used for 1 h at 37°C. Nuclei were stained with DAPI (1:10.000). For infected BMDMs, a double immunofluorescence staining was performed. Cultures were initially fixed with 0.05% glutaraldehyde and 3% paraformaldehyde. After permeabilization with Triton X-100, cells were incubated with *N. caninum*-infected mouse serum (1:2000) as a primary antibody (25) for 1 h at 37°C, followed by incubation with Alexa Fluor 488-conjugated goat anti-rabbit IgG (Life Technologies, Carlsbad, CA, USA) (1:750) for 1 h at 37°C. For macrophage staining, Alexa Fluor-594 Phalloidin (Life Technologies, Carlsbad, CA, USA) was used for 30 min at 37°C. Nuclei were stained with DAPI at 1:10000 in PBS. Images for each condition were obtained using an inverted fluorescence microscope (Nikon Eclipse TE200) equipped with a PCO.panda scientific CMOS camera. Imagining was performed at 40 ✕ or 100 ✕ magnification, and data processed through the Imaging Software NIS Elements (v. 5.30.04). Only linear adjustments to brightness and contrast were applied uniformly across each image, without altering the integrity or interpretation of the data.

### Humoral immune response in murine infections

*N. caninum* specific IgG1 and IgG2 serum levels were determined in female mice using ELISA as previously described (19). *N. caninum* soluble tachyzoite antigen was coated onto 96-well plates (0.125 μg/well), and ELISA was performed using a 1:100 dilution of serum samples. Peroxidase-conjugated anti-mouse IgG1 or IgG2a were used as secondary antibodies (1:5000; Southern Biotechnology, Birmingham, AL, USA). As controls, sera from mice experimentally infected with Nc-Spain7 and non-infected mice from previous experiments were used (5). Absorbance was measured at 405 nm for each plate, and results were expressed as a relative index percentage (RIPC) following the formula: RIPC = (OD sample – OD negative control)/(OD positive control – OD negative control) ✕ 100.

### Transcriptomic analysis

#### RNA library preparation and sequencing

BMDM were left uninfected or infected with *NcΔROP2* or WT strains for 48 hours pi before RNA extraction using miRNeasy Kit (Qiagen). Total RNA (500 ng per sample) was used to RNA-seq libraries using the NEBNEXT Ultra II Directional RNA Library Prep Kit (NEB #7760) and the Poly-A mRNA Magnetic Isolation Module (NEB #E7490), following the manufacturer’s protocol. Poly-A mRNA was purified using poly-T oligo-attached magnetic beads, fragmented under elevated temperature with divalent cations, and primed with random hexamers. First-strand cDNA synthesis was performed using reverse transcriptase and random primers, followed by second-strand synthesis incorporating dUTP to prevent amplification of the second strand. Double-stranded cDNA was purified using AMPure XP beads (Beckman Coulter, #A63881) to obtain blunt-ended fragments. A single ‘A’ nucleotide was added to the 3’ ends to facilitate adapter ligation. Adapter-ligated DNA was purified with AMPure XP beads and amplified with 11 cycles of PCR using unique dual index sequences. Final libraries, purified with AMPure XP beads, had an average fragment size of 299 bp, including index and adapter sequences. Sequencing was performed on the Illumina NextSeq 2000 platform (Illumina Inc, #20038897) using NextSeq 1000/2000 P2 Reagents (200 Cycles) v3 (#20046812). Libraries were pooled equimolarly (n = 20) based on Qubit and Bioanalyzer quantification and sequenced on one P2 flow cell. PhiX Control v3 (Illumina Inc, #FC-110-3001) was spiked into each run at 1% for quality control.

#### Dual RNA-seq data processing and bioinformatic analysis

FastQC software (v0.12.1) was used to perform quality control of raw sequencing reads. Raw reads were then pseudo-aligned to both *Bos taurus* (ARS_UCD1.3, Ensembl v111) and *N. caninum* Liverpool (ToxoDB v66) reference genome using Kallisto (v0.43.0) (26), with 100 bootstraps and —rf-stranded option specified. Downstream analyses were performed in R (v4.3.2). The transcript-level counts generated by Kallisto were aggregated to gene level using the tximport package (v1.30.0) (27) and differential expression analysis conducted separately for the host and parasite specific reads using DESeq2 (v1.42.0) (28). Differentially expressed genes (DEGs) were identified at a false discovery rate (FDR) threshold of <0.05.

For the host transcriptome, DEGs with an absolute log2Fold change value > 0.5 were selected. Host genes were converted to human orthologs using biomaRt package (v2.58.2) (29) for functional analysis. Kyoto Encyclopedia of Genes and Genomes (KEGG) (30) pathway enrichment analyses were performed using the clusterProfiler package (v4.10.1) (31). Gene set enrichment analysis (GSEA) (32) was conducted using clusterProfiler with the Hallmark gene sets downloaded from msigdbr (v7.5.1) (33), based on the ranked list of genes according to the DESeq2 statistic value. Gene sets with an FDR < 0.05 were considered significantly enriched. The DecoupleR package (v2.8.0) (34) was used to calculate Transcription Factor (TF) activity for each contrast based on statistical values from DESeq2, utilizing DoRothEA regulons (35). For the parasite transcriptome analysis, DEGs were mapped to their orthologs in the *T. gondii* ME49 reference genome from ToxoDB (v66) for functional analysis. The ggplot2 (v3.5.0), ComplexHeatmap (v2.18.0) (36), and ComplexUpset packages were used to visualize the results.

#### Parasite susceptibility to IFN-γ-mediated growth inhibition in bovine monocyte-derived macrophages

BMDMs were seeded in 24-well culture plates for 4 h then left-unstimulated or stimulated different concentrations of interferon gamma (IFN-γ) (0.1 and 10 ng/ml; Kingfisher Biotech Inc., St. Paul, MN, USA) for 24 h. The cells were then infected at an MOI of 0.5 with Nc-Spain7 or *NcΔROP2* tachyzoites. At 48 h or 60 h post infection, cells were lysed and stored at −80°C until DNA extraction. Tachyzoite burden was quantified by qPCR and expressed as the relative growth (%) compared to WT strain. For each IFN-γ concentration, BMDMs obtained from at least two independent experiments were used and 12 replicates per dose were analyzed.

#### Tachyzoite-bradyzoite stage conversion assay

To assess the expression of bradyzoite related genes, 6 well plates seeded with 3×10^6^ HFF cells per well were infected with tachyzoites at an MOI of 1. Conversion was induced by adding sodium nitroprusside (SNP) (70 μM) at 24 h pi and continued throughout the seven-day experiment as previously described (37). After total RNA isolation, cDNA was synthesized from 50 ng RNA using the NZY First-Strand cDNA Synthesis Kit (Nzytech, Portugal) following the manufacturer’s instructions. Specific primers for the bradyzoite- and tachyzoite- specific genes *NcSag4* and *NcSag1*, respectively, were used, as well as the 18S ribosomal RNA gene as housekeeping (**Supplementary Table S1**). NcSAG4 is a well-characterized bradyzoite-specific marker in *N. caninum* (37) while NcSAG1 is an immunodominant surface antigen specific to tachyzoites (38). qPCR was carried out using the 7500 FAST Real-Time PCR System (Applied Biosystems, Foster City, CA, USA) and GoTaq^®^ qPCR Master Mix (Promega, Madison, WI, USA). All samples were processed in duplicates. The results were expressed as fold induction using the 2^-ΔΔCt^ formula. –ΔΔCt was determined by first calculating the differences between the mean threshold cycle values of NcSAG4 or NcSAG1 and Nc18sR (normalizer) amplicons for each sample and then subtracting the baseline sample’s ΔCt (unstressed parasites from each day).

### Statistical analysis

Differences in mortality rates were evaluated using Fisher’s F test, while the Kaplan-Meier survival method was used to estimate the percentage of surviving animals at each time point. The log-rank (Mantel-Cox) test was used to compare survival curves and calculate the median survival time. Clinical sign scores and parasite burden between groups were analyzed using the Kruskal-Wallis test followed by Dunn’s multiple-comparison test. Litter size and antibody levels were compared using one-way ANOVA followed by Tukey’s multiple-comparison test, preceded by D’Agostino-Pearson test for normal distribution. Regarding cIR, parasitic vacuole size and proliferation data, a parametric one-way ANOVA test followed by Dunnett’s test for group comparisons was used. For mRNA levels, a student t test was performed to compare groups. Statistical significance was defined at *p* < 0.05 for all analyses. All statistical analysis were conducted using the GraphPad Prism v.7.0 software (San Diego, CA, USA).

## Results

### Successful construction of the *NcRop2* knockout and complemented strains

**Figure 1:**
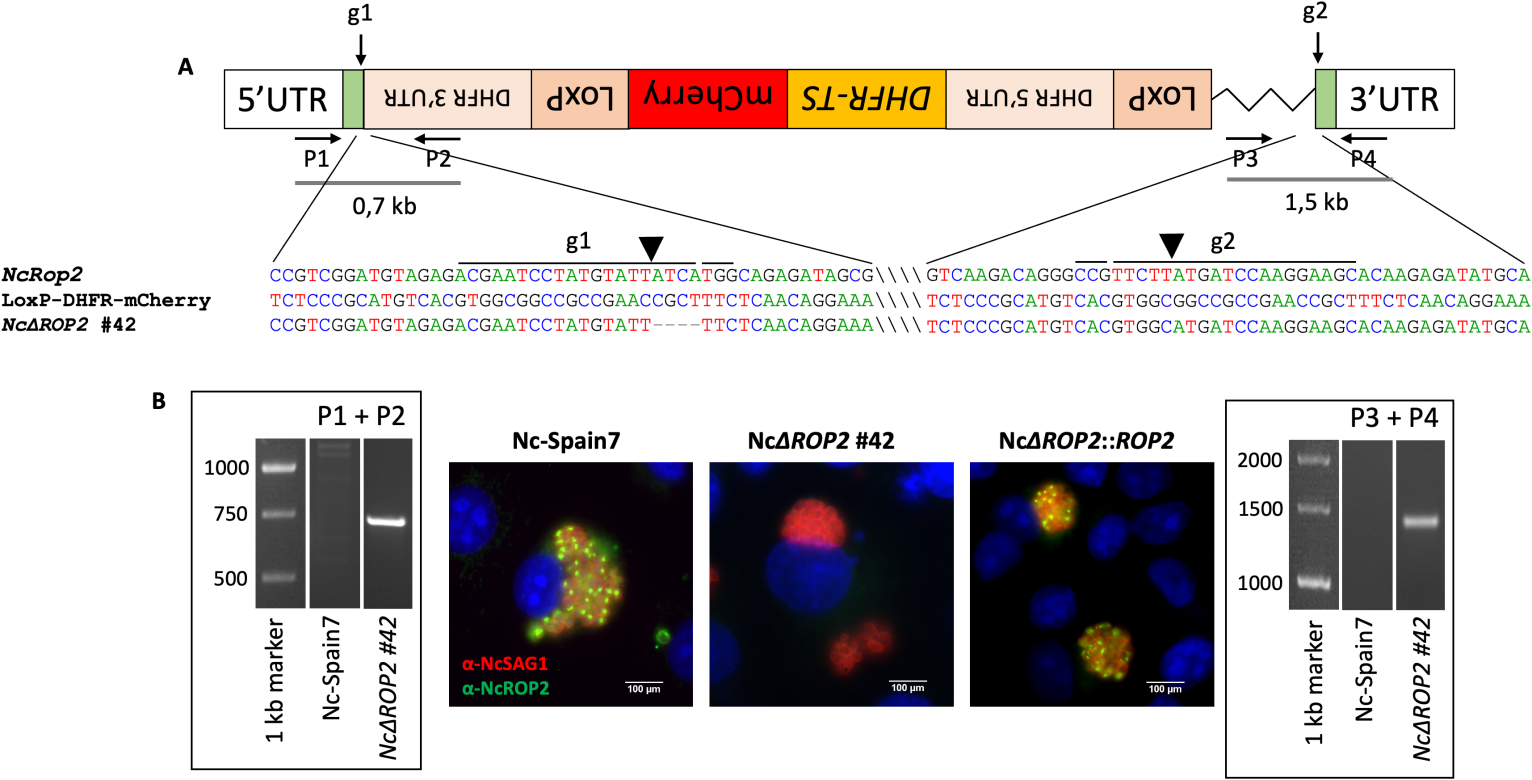
Construction of *N. caninum* NcΔROP2 #42 knockout strain. **(A)** Schematic representation of the *NcRop2* mutant clone that incorporated the donor template. The position and direction of primers (P1-P4) used for diagnostic PCRs are indicated by arrows. Diagnostic PCR demonstrate the integration of the donor template into the *NcROP2* locus (P1 + P2 and P3 + P4). Sequence alignments of the regions surrounding the cutting site are shown, comparing the KO clone with the *NcROP2* locus and the loxP-DHFR-mCherry plasmid. gRNA sites and protospacer adjacent motif (PAM) sequences are marked with lines above the sequences. Slashes within the sequences are used to visually separate distant unconnected regions. The LoxP-DHFR-TS-mCherry-LoxP cassette is shown in reverse orientation to reflect the actual direction of integration as confirmed by sequencing. **(B)** Immunofluorescence analysis of *NcΔROP2* parasites. MARC145 cells infected with different *N. caninum* strains were analysed by immunofluorescence. nuclei are stained with DAPI (blue), while the surface of the parasites is stained in red (NcSAG1). NcROP2 is labelled in green. The cutting sites are indicated by arrowheads. UTR, untranslated region; DHFR-TS, dihydrofolate reductase-thymidylate synthase.

To unravel the functional role of NcROP2, we efficiently generated two different *NcΔROP2* strain clones. The *NcΔROP2* #42 clone had most of the *NcRop2* coding region replaced by the *DHFR-TS* cassette (**Figure 1A**), while clone *NcΔROP2* #8 presented a 7 bp deletion at the 5’ end of the gene, generating a frameshift mutation. To validate the successful gene deletion, we performed PCR analyses using primers targeting the *NcRop2* flanking regions, as well as the *DHFR-TS* cassette (**Supplementary Table S1**). Our results showed the presence of amplified bands in the parasites with disruptions in *NcΔROP2* while being absent in the parental strain (**Figure 1B**). The gene disruption was also confirmed by Sanger sequencing and the absence of NcROP2 protein expression was evaluated by IFAT (**Figure 1B**).

To generate a complemented strain, we used the *NcΔROP2* #42 clone as the parental strain, as it had most of the *NcRop2* gene removed. We constructed complemented strains by reintroducing an exogenous copy of the target gene *NcRop2* into the UPRT locus via double homologous recombination, using overhangs in the *Nc*ROP2 repair template homologous to the 5’ and 3’ UTR region of the UPRT locus to enhance efficiency. Single clones of the *NcRop2* complemented strain were obtained by limiting dilution, and the expression of *Nc*ROP2 protein assessed by IFAT, with expression levels mirroring those observed in the Nc-Spain7 strain (**Figure 1B**).

Collectively, these data confirm the successful generation of *NcΔROP2* KO strains, as well as their corresponding complemented strain (*NcΔROP2*::*ROP2*).

### *NcΔROP2* shows attenuated virulence in mice

**Figure 2:**
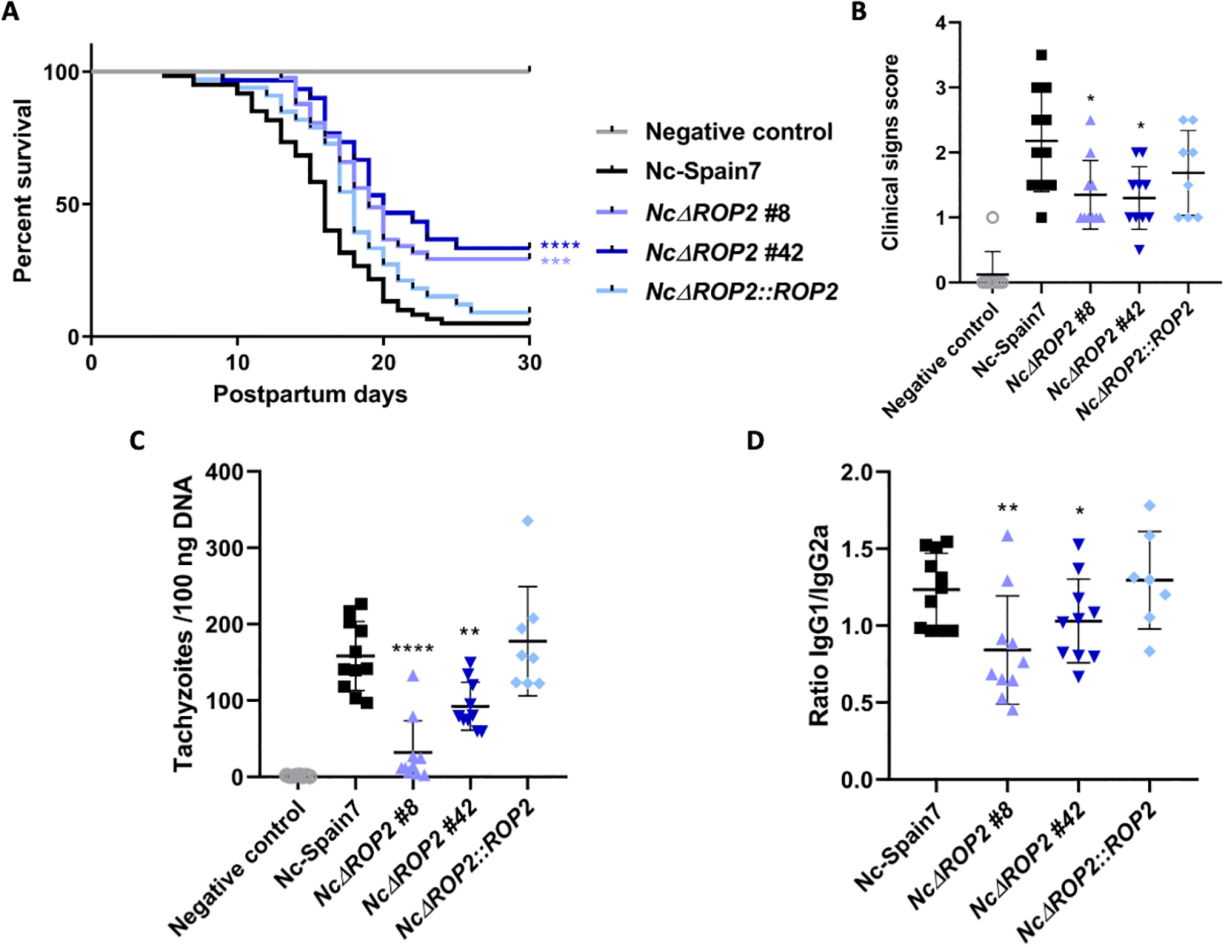
Effects on pregnant mice infected with *NcΔROP2.* **(A)** Kaplan-Meier survival curves of pups born to dams infected with *N. caninum*. Pregnant dams were infected with 10^5^ tachyzoites from different *N. caninum* strains on day 7 of gestation, and the survival of the offspring was monitored until day 30 post-partum. Each data point on the curve represents the percentage of surviving pups at a given day, with vertical steps indicating death events. Significant differences are denoted by asterisks (****ρ < 0.0001; ***ρ < 0.001; log-rank test). **(B)** Clinical signs in dams infected with *N. caninum* tachyzoites. Clinical signs were scored based on their detection and severity (0: no alterations; 1: ruffled coat; 2: rounded back; 3: severe weight loss; 4: nervous signs). Each dot represents an individual animal. Significant differences between groups are indicated by asterisks (*ρ < 0.05; Kruskal-Wallis, Dunn’s comparison post-test). **(C)** Cerebral parasite load in dams infected with *N. caninum* strains. Each dot represents individual values, while medians are indicated by horizontal lines. Parasite load is expressed as the number of parasites per 100 ng of host DNA. Significant differences between infected groups are indicated by asterisks (****ρ < 0.0001; **ρ < 0.01; Kruskal-Wallis, Dunn’s comparison post-test). **(D)** IgG1/IgG2a *Neospora*-specific antibody ratio at 30 days postinfection. Each dot represents the IgG1/IgG2a ratio, while the median and standard deviation for each group are shown by horizontal and vertical lines, respectively. Significant differences between groups are denoted by asterisks (**ρ < 0.01; *ρ < 0.05; one-way ANOVA Tukey’s comparison post-test).

In the *N. caninum* congenital murine model, pups born from dams infected with either of the *NcΔROP2* clones exhibited a significant increase in median survival times (19 and 20 days respectively) in comparison to the Nc-Spain7-infected group (16 days) (ρ < 0.01, log-rank test; **Figure 2A**) (**Table 1**). *NcΔROP2*-infected groups showed significantly lower mortality rate, with 33% and 28% of the pups surviving at the end of the experiment (day 30 pp) for clones #42 and #8, respectively, while all other infected groups reached postnatal mortality rates close to 100% (**Table 1**). Furthermore, the complementation of *NcΔROP2* led to a restoration of parasite virulence, as the offspring from the *NcΔROP2*::*ROP2* group showed no significant differences in median survival time nor mortality rate compared to those from the Nc-Spain7 group (ρ > 0.05, log-rank test). Statistical analysis revealed no significant differences in rates of pregnancy (ρ > 0.05, Fisher’s F test) or litter size (ρ > 0.05, one-way ANOVA) among mice infected with the different strains (**Table 1**).

**Table 1:** Impact of *N. caninum* infections on pregnant BALB/c dams and offspring.

Group	Fertility (%)^a^	Litter size^b^	Postnatal survival (%)^c^	Median survival time (days)^d^
Negative control	11/14 (78)	4.10 ± 2.11	43/43 (100)	>30
Nc-Spain7	11/18 (61)	3.00 ± 1.54	2/28 (7)	16
*NcΔROP2 #8*	10/20 (50)	6.83 ± 1.72	14/50 (28)	19
*NcΔROP2 #42*	10/18 (56)	4.60 ± 1.96	12/36 (33)	20
*NcΔROP2::ROP2*	8/18 (44)	4.25 ± 1.92	2/32 (6)	17

^a^Proportion of pregnant mice per group. ^b^Number of delivered pups per dam. ^c^Proportion of surviving pups at day 30 post-partum (%). ^d^Day post-partum at which 50% mortality was reached.

In the dams, clinical signs of neosporosis became evident starting from two weeks post infection. Initial manifestations included rough hair coat and apathy, followed by anorexia, inactivity, and eventually, neurological signs. Notably, groups infected with either of the *NcΔROP2* clones exhibited significantly milder clinical signs (ρ < 0.05, Kruskal-Wallis, Dunn’s comparison post-test; **Figure 2B**). Furthermore, as detailed in **Table 1**, lower parasite burdens were observed in dams infected with the *NcΔROP2* clones compared to those infected with the Nc-Spain7 wild type strain (**Figure 2C**). Similarly, in non-pregnant mice, a significant lower parasite burden was observed in those infected with the *NcΔROP2* clones compared to the Nc-Spain7 group (ρ < 0.01. Data not shown).

Significantly higher levels of specific anti-*N. caninum* IgG1 and IgG2a antibodies were detected in all infected groups compared to the negative control group (ρ < 0.0001, one-way ANOVA Tukey’s comparison post-test), confirming *N. caninum* infection (data not shown). Dams infected with the *NcΔROP2* clones exhibited a significantly lower IgG1/IgG2a ratio (ρ < 0.05; one-way ANOVA Tukey’s comparison post-test), while complementation of *NcRop2* restored the ratio to levels comparable to Nc-Spain7 (**Figure 2D**).

### Assessment of the *NcΔROP2 in vitro* phenotype in bovine macrophages highlights strain differences

Investigations of the consequences of the *NcROP2* deletion on the parasite lytic cycle in BMDMs were carried out using only the *NcΔROP2* #42clone, since both #8 and #42 exhibited a similar phenotype in *in vivo* experiments, and #42 was the parental strain for complementation. Additionally, the *NcΔROP2::ROP2* complemented strain was not tested because it displayed a phenotype similar to the WT Nc-Spain7 strain, further confirming that the observed differences were specifically due to *NcRop2* disruption.

**Figure 3:**
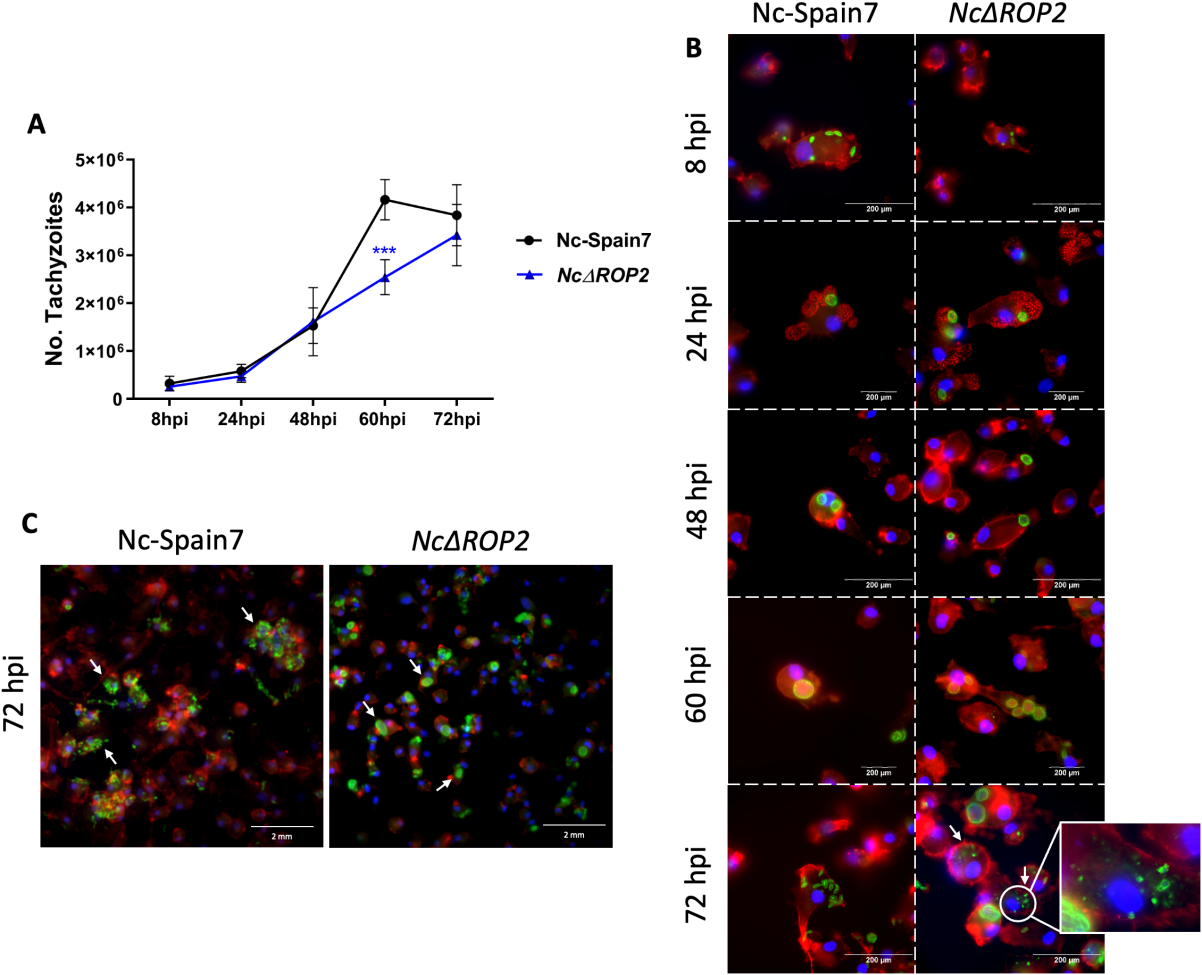
Analysis of parasite phenotype in BMDMs. **(A)** Proliferation kinetics of the parental strain Nc-Spain7 and the knockout strain *NcΔROP2* in BMDMs over time. The graph represents the proliferation kinetic using an MOI of 0.5:1, showing the average number of tachyzoites quantified by qPCR for each time point. The error bars indicate the standard deviation (SD). For each time point assayed, 12 replicates obtained from two independent experiments were used. Significant differences between the *NcΔROP2* and the Nc-Spain7 are denoted by asterisks (***ρ < 0.001, one-way ANOVA test, Dunnett’s comparison post-test). **(B)** Immunofluorescence staining images showing the progression of the lytic cycle in the Nc-Spain7 and KO strain over time. F-actin is stained in red, nuclei in blue and parasites in green. White arrows denote parasite degradation. **(C)** Immunofluorescence staining images at 72 hours pi showing the differences in egression. F-actin is stained in red, nuclei in blue and parasites in green. White arrows denote parasite egression in Nc-Spain7 and parasitophorous vacuoles in *NcΔROP2*.

The KO strain (*NcΔROP2* #42) initially displayed a proliferation pattern similar to that of the Nc-Spain7 strain, characterized by an exponential growth up to 48 hours pi. However, a notable divergence became evident beyond this point, when the *NcΔROP2* strain exhibited a significantly slower growth at 60 hours pi compared to the Nc-Spain7 parasites (ρ < 0.001) (**Figure 3A**).

Microscopic examination revealed that both the WT and KO strains successfully infected host cells by 8 hours pi, as intracellular parasites were already visible within BMDMs. By 24 hours pi, multiplication had begun, and parasitophorous vacuoles started forming, reaching their maximum size between 48 and 60 hours pi (**Figure 3B**). At this stage, no significant differences in the size of parasitophorous or percentage of infected macrophages were observed (data not shown). By 72 hours pi, host cells ruptured and tachyzoites were released in the Nc-Spain7 strain. In contrast, the *NcΔROP2* strain exhibited a slightly delayed egress, with a portion of parasites remaining intracellular longer than their WT counterparts, as can be seen by the presence of parasite vacuoles (**Figure 3C**). Additionally, an increased presence of vacuoles containing partially degraded tachyzoites or multinucleated complexes were observed (**Figure 3B**), suggesting that either the KO parasites were more prone to host-mediated degradation or that the egress process was incomplete. At 72 hours pi, some macrophages retained remnants of parasitic structures characterized by irregular morphology, loss of defined boundaries, and fragmented or diffuse staining patterns. These features are consistent with intracellular degradation processes previously described in *N. caninum* (39) and reinforce the idea that *NcΔROP2* mutants face challenges in maintaining their intracellular cycle and exiting efficiently.

### Immune modulation and host responses differ in *NcRop2*-deficient *N. caninum*

**Figure 4:**
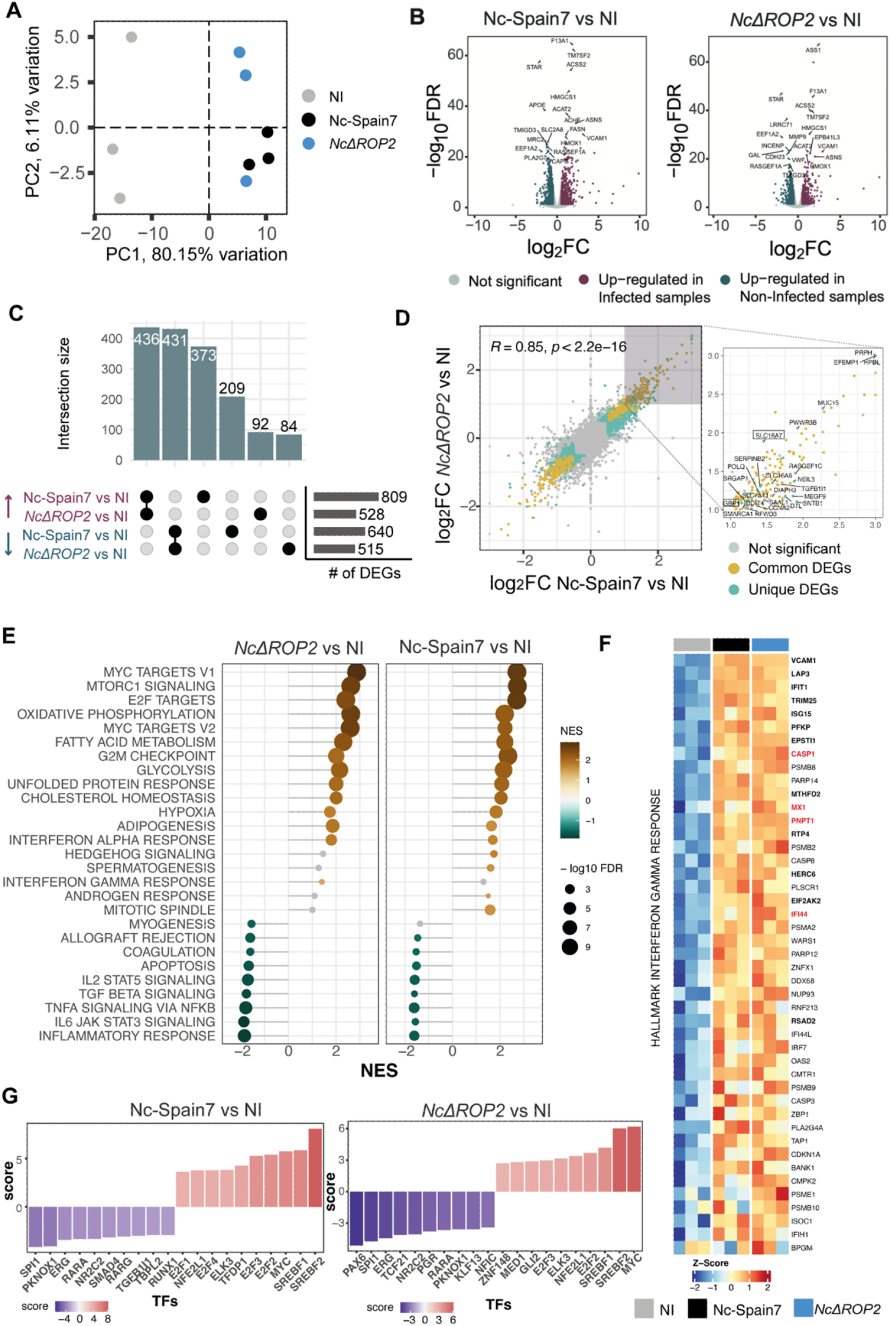
Differential host gene expression and pathway activation in response to *NcΔROP2* infection. **(A)** Principal Component Analysis (PCA) of RNA-seq data for host transcriptome data. Colors correspond to different parasite infections: non-infected (NI), Nc-Spain7, and *NcΔROP2*. **(B)** Volcano plots for Nc-Spain7 *vs* NI (left) and *NcΔROP2 vs* NI (right) infections. Genes are plotted based on their log2 fold-change (log2FC) and p-value. Upregulated genes are shown in pink, downregulated genes in blue and unchanged genes in grey. Top 10 differentially expressed genes (DEGs) are also highlighted. **(C)** An UpSet plot illustrating intersections of DEGs, highlighting upregulated and downregulated genes unique to Nc-Spain7, *NcΔROP2*, or shared across both conditions relative to NI controls. The horizontal bar plot provides a summary of the total number of up-regulated and down-regulated DEGs for each comparison. **(D)** Scatter plot comparing log2FC of DEGs between the Nc-Spain7 *vs*. NI and *NcΔROP2 vs*. NI comparisons. Each point represents an individual gene, with color indicating whether it is not significantly differentially expressed (grey), commonly differentially expressed across comparisons (yellow), or unique differentially expressed in one comparison (blue). Pearson correlation coefficient (R) and corresponding p-value are indicated. Unique genes for one of the comparisons are labelled on the right plot, with those inside a square being unique to the *NcΔROP2 vs*. NI comparison. **(E)** Gene Set Enrichment Analysis (GSEA) of hallmark pathways, with normalized enrichment scores (NES) displayed for *NcΔROP2* and Nc-Spain7 infections compared to NI controls. Positive enrichment (brown) and negative (green) are shown and bubble size corresponds to the number of genes associated with each pathway. **(F)** Heatmap showing z-score normalized expression of interferon gamma-related genes from core enrichment of GSEA. DEGs from both contrasts are marked in bold font, while red genes are unique to the *NcΔROP2 vs*. NI comparison. Values range from red (upregulation) to blue (downregulation) of key immune-related genes. **(G)** Transcription factor (TF) activity analysis based on RNA-seq data for *NcΔROP2* and Nc-Spain7 infections. Y axis reflects NES value for TF activity and color intensity represents the statistical significance of activity changes. Top 10 TF are shown.

Our results demonstrated that in the absence of NcROP2, parasites have attenuated virulence in mice and reduced growth in BMDM compared to the parental strain Nc-Spain7. To investigate the mechanisms underlying these phenotypic traits, we performed a comparative transcriptomic analysis of BMDMs infected with *NcΔROP2* and Nc-Spain7 strains. The RNA-seq analysis revealed significant transcriptional differences at both the host and parasite levels. Principal Component Analysis (PCA) of host expression data (**Figure 4A**) demonstrated distinct clustering between infected and non-infected samples, underscoring a strong transcriptional response to infection. Genes such as STAR, RASGEF1A and TMIGD3 were significantly downregulated in both comparisons (**Figure 4B**), pointing to the suppression of immune response and intracellular signaling related pathways. On the other hand, genes such as ACSS2, ACAT2, ASNS and HMGCS1, which are involved in lipid metabolism and cholesterol biosynthesis, were significantly upregulated, indicating a metabolic shift in infected cells.

In host transcriptional profiles (**Figure 4C**), *NcΔROP2*-infected macrophages exhibited 528 upregulated and 515 downregulated genes compared to non-infected cells (**Supplementary Table S2**). Of these, 92 upregulated and 84 downregulated genes were unique to *NcΔROP2* relative to non-infected cells. This unique set of DEGs suggests that these changes are due to the absence of ROP2 rather than the general effects of *N. caninum* infection. Interestingly, one of the unique DEGs for *NcΔROP2* infected cells is GBP1, which encodes a key interferon-stimulated gene (ISG) involved in host defense against intracellular pathogens. Despite these differences, the Pearson correlation coefficient (R = 0.85, p < 2.2e−16) between the log2FC values of Nc-Spain7- and *NcΔROP2*-infected macrophages indicates a strong overall similarity in host response patterns (**Figure 4D**).

To further explore these transcriptional changes, we conducted a functional enrichment analysis of DEGs (**Supplementary Figure S1**). Upregulated genes were primarily associated with pathways related to cell division, DNA replication and glycolysis, suggesting that both strains reprogram host metabolism to support parasite replication. Downregulated genes were predominantly related to immune processes, such as T cell activation, cytokine production and leukocyte differentiation, highlighting a shared strategy of immune evasion.

Next, Gene Set Enrichment Analysis (GSEA) was performed using the curated Hallmark gene set database (**Figure 4E**), to gain further insight into these transcriptional differences. *NcΔROP2*-infected BMDMs exhibited enrichment of gene sets related to metabolic pathways, including MYC targets, oxidative phosphorylation, and fatty acid metabolism. Conversely, BMDM infected with Nc-Spain7 showed enrichment in gene sets associated with cell cycle processes, such as E2F targets, G2/M checkpoint, and mitotic spindle, suggesting a focus on host cell cycle modulation.

Downregulated gene sets also revealed suppression of immune-related pathways in both strains, including inflammatory response, IL2 STAT5 signaling and TNFA signaling, underscoring their ability to inhibit pro-inflammatory and immune signaling pathways. Interestingly, interferon-related pathways, such as interferon-alpha (IFN-α) response and interferon-gamma (IFN-γ) response, were more strongly upregulated in *NcΔROP2*-infected BMDM, suggesting that while general immune activation was suppressed, interferon signaling pathways were more activated in the absence of ROP2.

To test the hypothesis that *NcΔROP2*-infected cells ineffectively suppress interferon signaling, we focused on host genes associated with IFN-γ response that change in response to *NcΔROP2* infection (**Figure 4F**). All DEGs exclusively upregulated in BMDM infected with *NcΔROP2* included ISGs such as *CASP1*, *MX1*, *PNPT1* and *IFI44*. These results support the notion that the deletion of *NcΔROP2* impairs the parasite ability to disrupt interferon signaling, a critical component of the host innate immune response.

To identify potential regulators driving the transcriptional changes observed in infected macrophages, DecoupleR was used to perform transcription factor (TF) enrichment analysis (**Figure 4G**). In *NcΔROP2*-infected BMDM, reduced activity was observed for immune-related TFs including SPI1 and KLF13, consistent with the downregulation of immune-related genes. By contrast, Nc-Spain7-infected BMDM displayed higher enrichment for TFs such as SREBF1/2, which regulate lipid metabolism, and the E2F family, key regulators of cell cycle progression. These findings align with the distinct transcriptional profiles induced by each strain.

**Figure 5:**
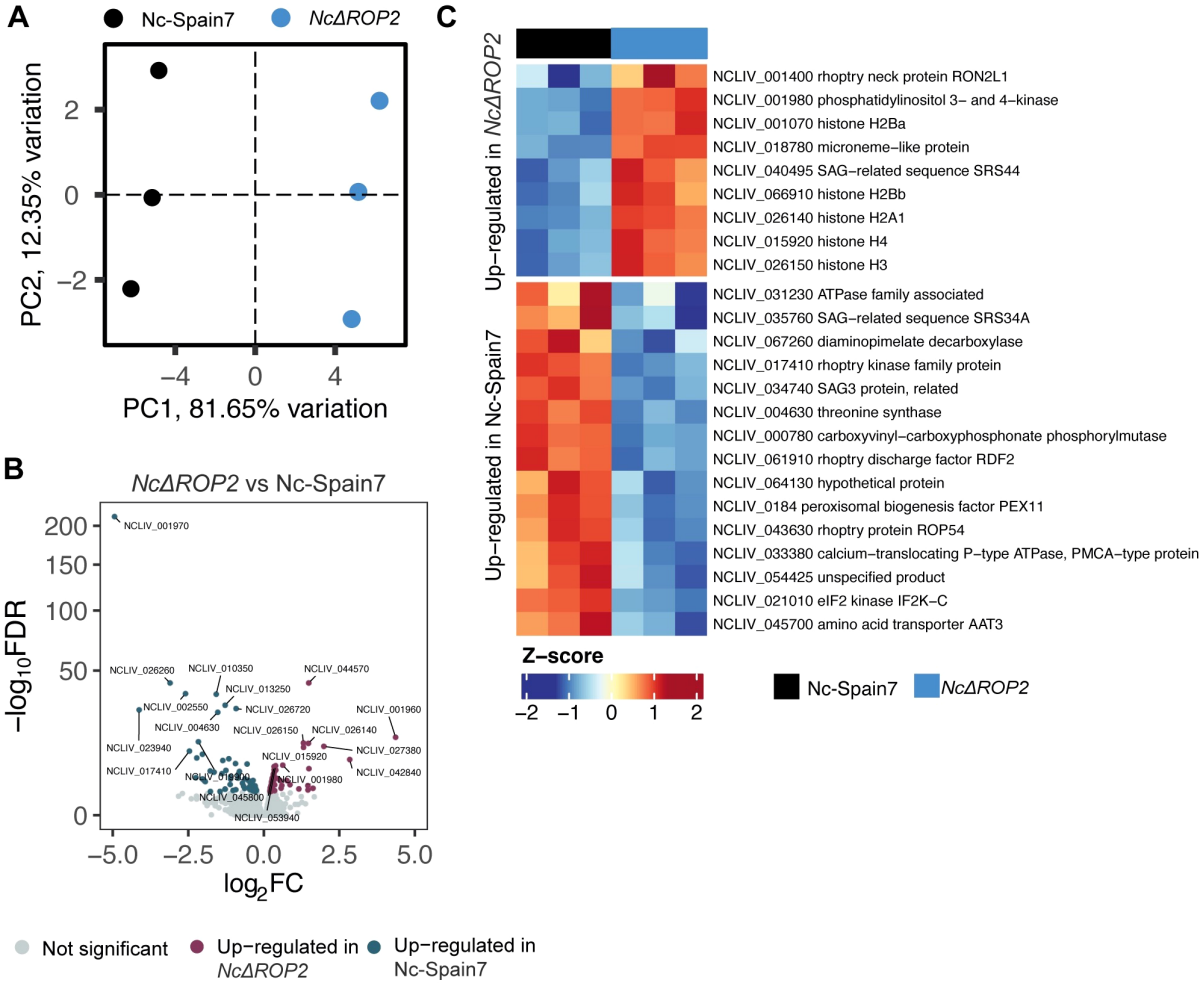
Parasite-specific transcriptomic changes induced by NcROP2 deficiency. **(A)** Principal Component Analysis (PCA) of RNA-seq data for parasite transcriptomes reveals sample clustering based on infection status. Colors correspond to different parasite strains: Nc-Spain7 and *NcΔROP2*. **(B)** Volcano plot depicting differentially expressed parasite genes (DEGs) between the *NcΔROP2* and Nc-Spain7 strains. Genes are plotted based on their log2 fold-change (log2FC) and statistical significance (-log10 FDR). Upregulated genes are shown in red, downregulated genes in blue, and non-significant genes in grey. Top 10 DEGs upregulated and downregulated are specified. **(C)** Heatmap illustrating the expression patterns of selected parasite specific DEGs between the *NcΔROP2* and Nc-Spain7 strains. Genes are clustered based on expression profiles, with rows representing individual genes and columns representing biological replicates. The color gradient represents normalized Z-scores of expression levels, with blue indicating lower expression and red indicating higher expression.

Analysis of parasite-specific transcripts also revealed significant differences between the KO and WT strains. PCA plots of parasite gene expression revealed clear separation between Nc-Spain7 and *NcΔROP2*, highlighting their transcriptional divergence (**Figure 5A**). In *NcΔROP2*, 84 genes were upregulated, while 63 genes were downregulated in Nc-Spain7 (**Figure 5B**) (**Supplementary Table S3**). Among the upregulated genes in *NcΔROP2* (**Figure 5C**) were histones such as H2A and H2B, suggesting changes in processes like chromatin remodeling and stress response. Additionally, bradyzoite-related genes, including RON2L1 and SRS44, were upregulated in *NcΔROP2*, suggesting a transcriptional shift toward a latent or stress-adapted stage, likely reflecting the parasite efforts to adapt to the absence of ROP2.

By contrast, *NcΔROP2* parasites displayed downregulation of key genes involved in host cell interaction, including rhoptry proteins (e.g., RDF2) and surface antigens (e.g., SAG3 and SRS34A). These molecules are critical for host cell manipulation and invasion, and their reduced expression highlights the functional consequences of ROP2 deletion. Collectively, these results illustrate the distinct transcriptional consequences in the absence of *NcROP2* and how the parasite adapts to this genetic landscape.

### Functional analyses confirm low sensitivity to IFN-γ and enhanced bradyzoite conversion in *NcΔROP2* parasites

**Figure 6:**
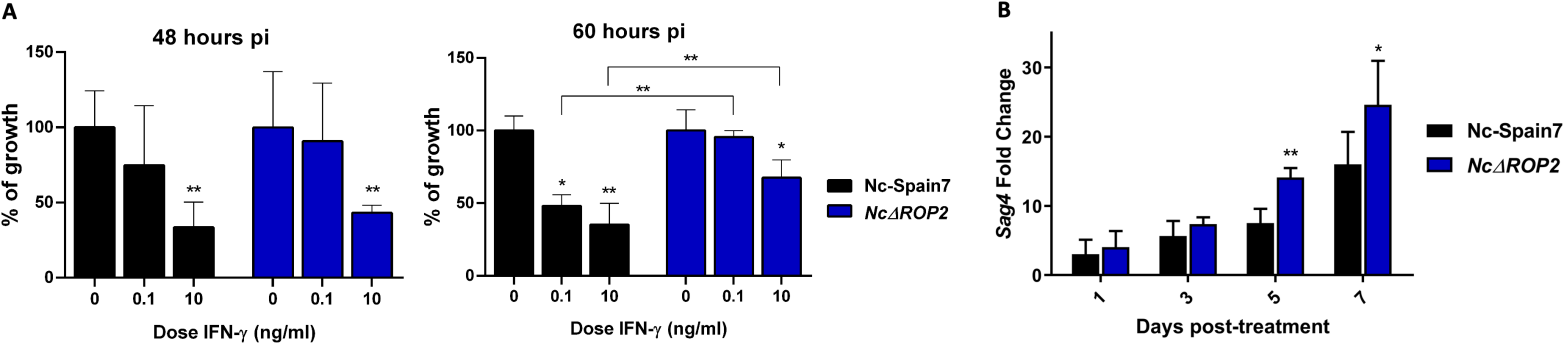
**(A)** Parasite growth of the parental strain Nc-Spain7 and the *NcΔBPK1* and *NcΔROP2* strains in BMDMs unstimulated or stimulated with different IFN-γ doses. The column graphs represent the parasite burden in BMDMs at 48 hours pi or 60 hours pi, respectively. For each point, BMDMs obtained from at least two independent experiments were used and 12 replicates per dose were analyzed. Error bars indicate the standard deviation (SD). Significant differences between each point compared to naïve BMDM are denoted by asterisks (*ρ < 0.05; **ρ < 0.01, one-way ANOVA test, Dunnett’s comparison post-test). Error bars indicate standard deviation (SD). **(B)** Expression levels of bradyzoite-specific gene Nc*Sag4*. Three independent experiments were performed. Gene expression was determined by RT-PCR over time of a stage-conversion assay by adding sodium nitroprusside. Fold induction is referred to untreated parasites and calculated by the 2^-ΔΔCt^ formula. Error bars indicate the standard deviation (SD). Significant differences between groups at same time points are each denoted by asterisks (*ρ < 0.05; **ρ < 0.01; Fisher’s F test).

To validate the transcriptomic results, we performed two independent assays: one to confirm the host transcriptomic findings and another to validate the parasite-related results. Since our transcriptomic analysis revealed differential activation of IFN-related pathways in BMDMs infected with *NcΔROP2*, we assessed their susceptibility to IFN-γ at both 48 hours pi and 60 hours pi (**Figure 6A**). At 48 hours pi, both Nc-Spain7 and *NcΔROP2* exhibited a similar dose-dependent reduction in parasite growth, with significant inhibition observed at 10 ng/ml of IFN-γ (ρ < 0.01, one-way ANOVA test, Dunnett’s comparison post-test). However, at 60 hours pi, while Nc-Spain7 continued to show a clear dose-dependent inhibition, *NcΔROP2* displayed a marked resistance to IFN-γ, with only minimal inhibition at the highest concentration (around 30%). Additionally, direct comparisons between Nc-Spain7 and *NcΔROP2* at the same IFN-γ doses revealed significant differences at 60 hours pi (ρ < 0.01, one-way ANOVA test, Dunnett’s comparison post-test), highlighting the impact of *NcRop2* deletion on parasite susceptibility to IFN-γ.

To further validate the transcriptomic findings that suggested an overexpression of bradyzoite-related genes in the *NcΔROP2* KO strain, we performed a tachyzoite-to-bradyzoite conversion assay and measured the *NcSag4* bradyzoite-specific gene expression at different time points following SNP stimulation (1, 3, 5, and 7 days) (**Figure 6B**). Over the course of the assay, *NcSag4* transcript levels progressively increased in both groups. However, significant differences were observed between the two groups at five and seven days post-infection, with a higher fold change in the *NcΔROP2* parasite (ρ < 0.01-0.05; Fisher’s F test). By contrast, *NcSag1* mRNA expression remained stable throughout the assay, with no significant differences between the initial and final time points for either of the strains (data not shown).

## Discussion

Numerous parasite proteins, particularly those secreted by rhoptries and dense granules, have been identified as virulence factors in apicomplexan parasites, as they play essential roles in host cell manipulation, immune modulation and nutrient acquisition, thereby promoting parasite survival and proliferation (3–5, 40). Among these parasite effectors, the ROP2 family represents one of the largest and best studied group of rhoptry proteins in *T. gondii* and includes protein kinases and pseudokinases that have been identified as virulence factors (41, 42). However, the role in the virulence of *N. caninum* is unknown. In this study, we successfully generated a *NcΔROP2* KO strain and used two different clones to investigate the role of this gene in *N. caninum* virulence. The first clone, *NcΔROP2* #42, had most of the *NcRop2* coding region deleted, ensuring full gene disruption. On the other hand, clone *NcΔROP2* #8 involved a targeted cut at the 5’ end of the gene, potentially allowing for the expression of nonfunctional protein fragments. The reason for studying two independent KO clones was to confirm that the observed phenotypic effects were specifically due to the loss of NcROP2 rather than off-target effects of genome editing (43). The disruption of *NcRop2* in both clones resulted in a significant virulence reduction in a well-established murine model of neosporosis, as evidenced by a longer survival time and reduced mortality rates in the offsprings, milder clinical signs and lower cerebral parasite burden in dams. This aligns with previous studies investigating the role of other virulence factors in *N. caninum*, particularly ROP and GRA proteins. For example, NcROP5 KO strains exhibited a markedly reduced cerebral parasite burden and an increased survival rate in infected mice (4). Similarly, the deletion of NcROP16 resulted in slower intracellular growth and reduced parasite proliferation in murine models (3). However, despite their similarities, an important distinction in our study is the choice of the WT strain. Previous studies on NcROP5 and NcROP16 used a milder-virulence isolate Nc-1 as reference. This difference in WT background could influence the extent of virulence attenuation observed in KO strains, as Nc-1 exhibits inherently lower pathogenicity than Nc-Spain7. Nonetheless, the comparable trends across studies reinforce the essential role of these virulence factors in modulating disease severity. Our study uses Nc-Spain7 as reference, the same WT strain used in previous experiments with the NcGRA7 and NcROP40 KO strains. These two KO strains demonstrated a significant attenuation of virulence, as evidenced by milder clinical signs and lower brain parasite loads in infected dams (5), however, the *NcΔROP2* strain exhibited an even greater attenuation of virulence compared to both NcGRA7 and NcROP40 KO strains.

In our study, the WT phenotype was rescued in the NcROP2 complemented strain, confirming that the observed effect in parasite virulence was due to the absence of ROP2. Regarding the humoral immune response, females infected with the *NcΔROP2* strain showed a significantly lower IgG1/IgG2a ratio, suggesting a polarization toward a Th1 response compared to those infected with Nc-Spain7. This finding is relevant, as Th1 response is more effective in controlling *N. caninum* infection (44, 45). Complementation of ROP2 restored the IgG1/IgG2a ratio to similar levels than those from the Nc-Spain7 group, further highlighting the role of ROP2 in modulating the host immune response. Altogether, our results demonstrate that NcROP2 is a parasite virulence factor that influences the severity of disease but are not essential for transmission or survival (46).

The absence of NcROP2 could impair the invasion and replication capacity of mutant parasites or interfere with mechanisms involved in immune response evasion or stimulation. To further investigate the role of NcROP2 in the parasite lytic cycle in its natural host, we assessed the growth of *NcΔROP2* KO tachyzoites in a well-established BMDMs model, which can provide information on the proliferation capacity, immune response modulation, and host transcriptome alterations between strains (6, 47). The absence of differences in parasite burden during the early hours in the KO strain suggests that once inside the host cell, *NcΔROP2* tachyzoites are still able to replicate efficiently during the initial phase of infection, which could be explained by the parasite division pattern. At this early stage (before 48 h), both the KO and WT strains may be replicating at similar rates, albeit in smaller numbers due to binary fission. It is only after 48 h, when exponential growth starts, that the differences between the strains, particularly related to NcROP2 role in replication and egress, become more apparent. By 72 hours pi, while Nc-Spain7 tachyzoites successfully exited host cells, *NcΔROP2* parasites exhibited a delay in egress, accompanied by an accumulation of degraded intracellular tachyzoites. Similar outcome has been described under stress conditions, such as exposure to bumped kinase inhibitors, where parasites failed to exit host cells and displayed a mixed expression of tachyzoite and bradyzoite markers (48). The persistence of parasitic remnants within BMDMs at this time point further supports the idea that NcROP2 may play a role in ensuring successful exit from the host cell, possibly by modulating vacuolar stability or host signaling pathways required for timely rupture. This suggests that the deletion of NcROP2 affects egress rather than intracellular multiplication within the vacuole. These findings align with previous reports indicating that NcROP2 protein is required for the egress phase. Specifically, higher NcROP2 mRNA levels were observed in recently invaded tachyzoites and at 56 hours pi, a critical time point when tachyzoites undergo egress to infect new host cells (13). This pattern supports the hypothesis that NcROP2 gene expression follows a “just-in-time” regulatory mechanism, wherein genes are transcribed only when their biological function becomes essential for parasite survival and propagation (49, 50). *In vivo*, the deficiency of a key protein involved in egress could result in reduced dissemination and a less severe infection, as observed in the murine model. Furthermore, it is possible that the reduced egress ability of the *NcΔROP2* KO strain could stress the parasite, causing it to enter a less replicative phase within the vacuole, thereby increasing its survival. In this line, the transcriptomic analysis revealed an upregulation in *NcΔROP2* of parasite genes associated with chromatin remodeling (e.g., histones H2A and H2B) and stress adaptation, such as RON2L1 and SRS44. This suggests a shift toward a latent or stress-adapted bradyzoite stage (39, 51–53), which may indicate that the deletion of NcROP2 disturbs the regulation of the parasite cell cycle. Consequently, in the absence of ROP2 parasites may favor persistence in the bradyzoite stage as a survival strategy when it is unable to egress and infect new cells. Indeed, when we assessed the capacity to transition to the bradyzoite stage an increase in the bradyzoite-specific *NcSag4* expression levels was observed in the *NcΔROP2* strain. However we did not observe a significant decrease in the tachyzoite-specific *NcSag1* expression levels during the assay in either strain. This could be explained by the gradual nature of the tachyzoite-to-bradyzoite conversion, where individual parasites within a vacuole may simultaneously express both tachyzoite and bradyzoite specific antigens until conversion is fully completed (54).

Host-transcriptomic analysis of Nc-Spain7 and *NcΔROP2-*infected cells strains provided valuable insights into the molecular mechanisms underlying host-pathogen interactions. Although the deletion of *NcRop2* resulted in subtle changes, as expected from the modification of a single gene, these changes significantly impacted parasite virulence and host immune responses. The high Pearson correlation between the two strains highlights the overall similarity of their transcriptional profiles, but some differences revealed how NcROP2 influences host and parasite adaptations during infection. DGEA revealed key host genes that are commonly upregulated or downregulated in both strains. Downregulated host genes, including STAR, RASGEF1A, and TMIGD3, all related to immune suppression mechanisms. The suppression of STAR disrupts cholesterol metabolism (55, 56), reducing macrophage activation (57), while RASGEF1A and TMIGD3 downregulation limits antigen presentation and macrophage differentiation into pro-inflammatory states (58, 59). By contrast, upregulated genes such as ACSS2, ACAT2, ASNS, and HMGCS1 reflect metabolic reprogramming in infected macrophages, supporting the parasite replication demands (60, 61).

Pathway enrichment analyses further illustrate the strategies employed by Nc-Spain7 and *NcΔROP2* to modulate host responses. Nc-Spain7 infection prominently enriched pathways associated with cell division and DNA replication. By driving host cell proliferation, Nc-Spain7 manipulates the intracellular environment to create a stable, nutrient-rich niche for parasite replication (62, 63). This contrasts with *NcΔROP2*-infected BMDM, where these pathways are less active, reflecting the KO strain reduced ability to control host cell cycle processes. The diminished control over cell division in *NcΔROP2* infections may lead to enhanced macrophage activation, as non-proliferative states are often associated with inflammatory phenotypes (64, 65). The TF activity analysis supports these transcriptomic findings. In Nc-Spain7-infected BMDM, higher activity of SREBF1/2 and the E2F family emphasized enhanced lipid biosynthesis and cell cycle regulation. SREBF1/2, as master regulators of cholesterol and fatty acid biosynthesis (66–68), align with the upregulation of genes such as HMGCS1 and ACAT2, highlighting Nc-Spain7 ability to manipulate host lipid metabolism to support parasite replication (60). Similarly, the E2F family activity correlates with enriched cell cycle pathways, reflecting Nc-Spain7 focus on maintaining host cell proliferation (63). In addition, the up-regulation of E2F transcription factors, such as E2F3, could be associated to the inhibition of the immune response observed in infected cells (69).

Apart from these differences in cell cycle processes between strains, notable variations in interferon signaling were observed. While both strains downregulated inflammatory pathways, such as TNFα signaling via NF-κB and IL2/STAT5 signaling, *NcΔROP2*-infected BMDM exhibited significantly stronger upregulation of interferon responses, particularly IFN-α and IFN-γ. This was accompanied by increased expression of ISGs, including IFI44, CASP1, MX1, PNPT1 or GBP1, reflecting heightened defenses in the absence of NcROP2 (70–72). GBP1 is known to enhance microbial activity by promoting inflammasome activation, ultimately restricting parasite survival (73, 74). The upregulation of GBP1, as well as other ISGs genes, in *NcΔROP2*-infected BMDM suggest an increase susceptibility of the KO mutant strain to host immune defenses, potentially due to impaired evasion mechanisms. This heightened susceptibility could contribute to the progressive degradation of parasites observed at later stages of the lytic cycle.

In light of these results, we evaluated the growth of the strains under immune pressure by IFN-γ. Although the understanding in *N. caninum* is limited, the closely related parasite *T. gondii* is known for developing several mechanisms to counteract IFN-γ-induced host defenses (75). These mechanisms include the secretion of effector proteins from rhoptries and dense granules, which co-opt host transcription and signaling pathways to manipulate host cell response to inflammatory signals (76–78).

In our study, both Nc-Spain7 and *NcΔROP2* showed a similar dose-dependent reduction in parasite burden at 48 hours pi, indicating that early-stage replication is equally susceptible to IFN-γ, similar to what has been observed in previous KO mutants in *N. caninum* (6). However, at 60 hours pi, the *NcΔROP2* strain displayed significantly lower susceptibility to IFN-γ compared to the WT strain suggesting that *NcRop2* deletion alters the parasite response to immune pressure. This result was somewhat unexpected, considering the increased expression of ISGs in *NcΔROP2*-infected cells, which would typically indicate a more robust antiparasitic environment. One possible explanation is that the *NcΔROP2* mutant may be in a metabolically less active or more differentiated state, such as an early transition to the bradyzoite stage, making it less susceptible to IFN-γ-dependent pathways that typically target rapidly replicating tachyzoites. The expression of bradyzoite-specific proteins may further contribute to this resistance, potentially explaining why the *NcΔROP2* mutant shows reduced susceptibility to IFN-γ, particularly at later stages of infection when bradyzoite-related pathways might be more active. Interestingly, similar findings have been reported in *T. gondii*, where parasites infecting IFN-γ-stimulated bovine macrophages primarily expressed bradyzoite-associated genes, reinforcing the idea that stress factors can drive developmental transitions (79).

Overall, the absence of NcROP2 in *N. caninum* appears to drive a metabolic and developmental shift that reduces replication and virulence. The transition likely limits parasite spread within the host, explaining the milder clinical signs and lower parasite burden observed in infected dams. Additionally, the activation of host immune signaling pathways enhances the ability of BMDMs to control the parasite, further contributing to the attenuated phenotype of *NcΔROP2*. This study underscores the crucial role of NcROP2 in promoting parasite replication and immune evasion during acute infection. Its deletion not only impairs proliferation but also facilitates an adaptive shift to a more dormant state, potentially as a survival strategy under stress pressure. These findings provide valuable insights into the interplay between parasite virulence, immune evasion, and host adaptation, highlighting NcROP2 as a key factor in *N. caninum* pathogenesis. Future studies should explore its molecular interactions with host immune pathways, which could inform novel therapeutic or vaccine strategies against neosporosis.

## Data Availability

The datasets presented in this study can be found in online repositories. The names of the repository/repositories and accession number(s) can be found below: https://www.ebi.ac.uk/ena, ERP169701.
