# Supplementary material for: NcROP2 deletion reduces Neospora caninum virulence by altering parasite stage differentiation and hijacking host immune response
Source: Front Immunol. 2025 Aug 12;16:1617570. doi: 10.3389/fimmu.2025.1617570 (PMC12379656; doi:10.3389/fimmu.2025.1617570)
Supplement: Supplementary file 1 [file DataSheet1.pdf]

Table 1 Supplementary. Primers used in the present work

Name	Sequence (5' – 3')	Description	Reference
ROP2 gRNA 1	TCTCTGCCATGATAATACAT	Disrupt ROP2 gene in 5' and 3' terminal ends	Present work
ROP2 gRNA 2	GCTTCCTTGGATCATAAGAA		
ROP2 Fw (P1)	AGCAAGAAGAGGTCGAGCAA	Check correct deletion/complementation of NcROP2	Present work
ROP2 Rv (P4)	TATTCCGTGTGCCTTGTGTG		
DHFR Fw (P2)	TCTGGCAGGCTACAGTGACA	Check correct deletion of the gene of interest	(Arranz-Solis et al., 2018)
DHFR Rv (P3)	GCCTGGTATCTTTATAGTCC		
pSS013 Fw	CAAATGGCGACCTGCAGAGG	Check correct ligation of gRNA into pSS013 plasmid	Present work
UPRT gRNA	GCAGGAGGAAAGCATTCTGC	Disrupt UPRT gene in 5' terminal end	(Rico-San Roman et al., 2022)
UPRT Fw1	tctagaggatccccgggtacCCAGCTGGTACTCTAGAAC	Amplification of the upstream flanking area from the Cas9 cutting site. sequence binding to the pUC19 plasmid is shown in lowercase	Present work
UPRT Rv ROP2	ttctgtaccaacggaccaaaCTGCTCAGTTGTCGCGTATC	Amplification of flanking area 5' of the Cas9 cutting point. Region that anneals with NcROP2 in lowercase	Present work
UPRT Fw2	TACCGCCAGGTAATCCTTC	Amplification of flanking area 3' of the Cas9 cutting point. Region that anneals with pUC19 in lowercase	Present work
UPRT Rv	agtgaattcgagctcggtacGGCAGACAGAATTGAAGAC		
ROP2 Fw2	TTTGGTCCGTTGGTACAGA	Generation of the exogenous copy of NcROP2 with UTR regions. Region that anneals with UPRT in lowercase	Present work
ROP2 Rv2	agaaggattacctggcggtaATCAGAGCCCGATGCCGCA		
28S Fw	TGCCATGGTAATCCTGCTCA	qPCR	(Collantes-Fernandez et al., 2002)
28S Rv	CCTCAGCCAAGCACATACACC		
Nc5 Fw	ACTGGAGGCACGCTGAACAC	qPCR	(Collantes-Fernandez et al., 2002)
Nc5 Rv	AACAATGCTTCGCAAGAGGAA		
18S Fw	GATACAGAACCAACCCACCTTCC	RT-qPCR	(Risco-Castillo et al., 2011)
18S Rv	AGACCGAAGTCAAACGCGATC		
NcSag4 Fw	GATTTCAAGAAGCCGCTGGA	RT-qPCR	(Fernandez-Garcia et al., 2006)
NcSag4 Rv	TGAGAACTTGTGTGTCGCCTGTT		
NcSag1 Fw	CGGTGTCGCAATGTGCTCTT	RT-qPCR	(Fernandez-Garcia et al., 2006)
NcSag1 Rv	ACGGTCGTCCCAGAACAAAC		
