# Supplementary material for: NcROP2 deletion reduces Neospora caninum virulence by altering parasite stage differentiation and hijacking host immune response
Source: Front Immunol. 2025 Aug 12;16:1617570. doi: 10.3389/fimmu.2025.1617570 (PMC12379656; doi:10.3389/fimmu.2025.1617570)
Supplement: Supplementary file 2 [file DataSheet2.pdf]

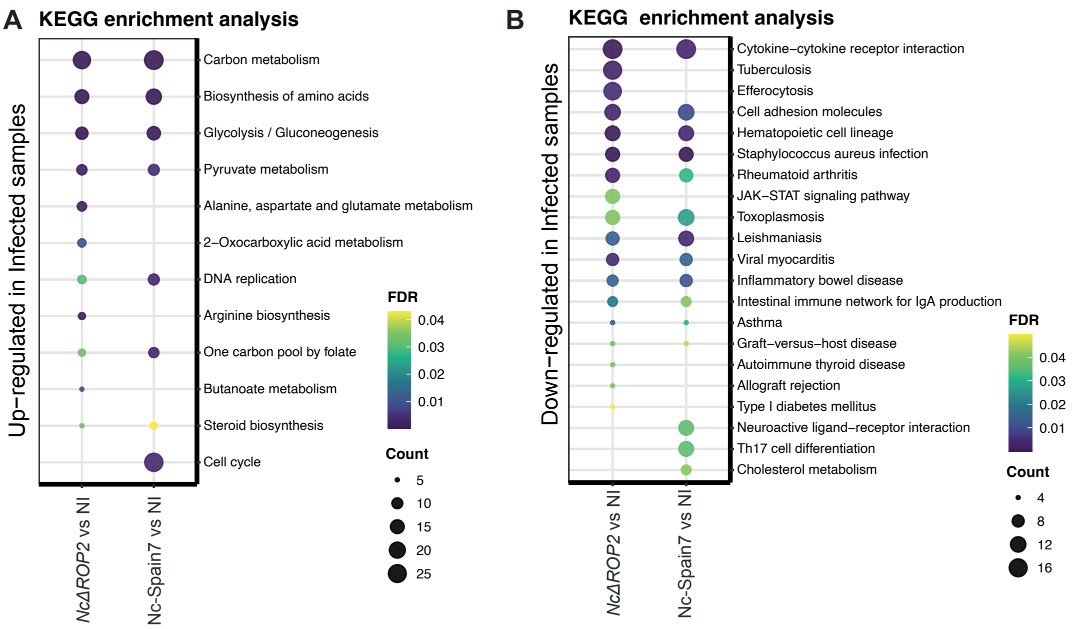

Supplementary Figure S1. Enrichment analysis of host genes in response to parasite infection. KEGG enrichment analysis for up-regulated DEGs (A) or down-regulated DEGs (B) in Nc-Spain7 and NcΔROP2 infections. Bubble plot shows the significant (FDR < 0.05) enriched Kyoto Encyclopedia of Genes and Genomes (KEGG) terms for DEGs, with the y-axis listing specific biological processes impacted by infection. Bubble colour represents the false discovery rate (FDR), ranging from green (higher FDR) to purple (lower FDR), while bubble size reflects the number of DEGs associated with each term.
